# Auxin-regulated timing of transition from vegetative to reproductive growth in rapeseed (*Brassica napus* L.) under different nitrogen application rates

**DOI:** 10.3389/fpls.2022.927662

**Published:** 2022-09-09

**Authors:** Pengfei Hao, Baogang Lin, Yun Ren, Hao Hu, Bowen Xue, Lan Huang, Shuijin Hua

**Affiliations:** ^1^Institute of Crops and Nuclear Technology Utilization, Zhejiang Academy of Agricultural Sciences, Hangzhou, China; ^2^Huzhou Agricultural Science and Technology Development Center, Huzhou, China; ^3^Institute of Digital Agriculture, Zhejiang Academy of Agricultural Sciences, Hangzhou, China

**Keywords:** auxin biosynthesis, floral meristem, nitrogen application, shoot apical meristem, yield

## Abstract

Accelerating the differentiation of floral meristem (FM) from shoot apical meristems (SAM) which determines the conversion from vegetative to reproductive growth is of great significance for the production of rapeseed (*Brassica napus* L.). In this research, the mechanisms of different nitrogen (N) application rates (low N, N1; normal N, N2; and high N, N3) on different FM development stages triggering the regulation of FM differentiation genes through the auxin biosynthetic and signal transduction were investigated. We found that the stage of FM differentiation, which was identified through a stereomicroscope and scanning electron microscope, came 4 and 7 days earlier under high N rate than under normal and low N levels, with the seed yield increased by 11.1 and 22.6%, respectively. Analysis of the auxin and its derivatives contents showed that the main biosynthesis way of auxin was the indole acetaldehyde oxime (IAOx) pathway, with 3-Indole acetonitrile dramatically accumulated during FM differentiation. At the same time, an obvious decrease of IAA contents at each FM differentiation stage was detected, and then gradually rose. Results of the expression of genes involved in auxin biosynthesis, auxin signaling transduction, and FM identification under five FM differentiation stages and three nitrogen application rates showed that genes involved in auxin biosynthesis were regulated before the FM differentiation stage, while the regulation of FM identity genes appeared mainly at the middle and later periods of the five stages, and the regulation level of genes varied under different N rates. Taken together, a high nitrogen rate could accelerate the initiation of FM differentiation, and auxin involved a lot in this regulation.

## Introduction

Appropriate timing of the transition from vegetative to reproductive growth is highly correlated with crop flowering time and yield (Narnoliya et al., 2019). The morphologic landmark of this change is a protrusion in the peripheral zone, which is called flower primordium (Carles and Fletcher, 2003). The differentiation of flower primordia derives from a population of cells called shoot apical meristem (SAM) (Tooke and Battey, 2003). The structure of the SAM had been elucidated in many plant species, which includes the central zone containing the stem cells, the peripheral zone initiating primordia, and the rib zone producing the internal part of the stem (Vernoux et al., 2010).

Extensive genetic studies identified many important genes controlling the transition from vegetative to reproductive growth. One of the regulating pathways of SAM differentiation is a system centered on a transcription factor WUSCHEL (WUS) (Hamada et al., 2000). The *WUS* signaling pathway was negatively regulated by *CLAVATA3* (*CLV3*), which encodes a small secreted signaling peptide (Xin et al., 2017). During SAM differentiation, the opposite roles between *CLV3* and *SHOOTMERISTEMLESS* (*STM*) were found (Nidhi et al., 2021). A combination of *STM* and *CUP-SHAPED COTYLEDON* (*CUC*)*1* family prevents new organ initiation (Balkunde et al., 2017). On the other hand, some key genes such as *LEAFY* (*LFY*) promote the outgrowth of flower primordium with the interaction of *WUS* or *AGAMOUS* (*AG*) (Traas, 2018). Therefore, the equilibrium between these two opposite gene systems is the main endogenous force for SAM differentiation. Other regulation systems for SAM differentiation were also identified. For example, post-transcriptional regulation via microRNAs (miRNAs) such as miR165/166 had important roles in SAM differentiation (Zhou et al., 2015). The most difficulties of these regulation systems are how the regulatory network is coordinated.

Among the mechanisms of feedback loops to sustain and restrict stem cell activities, one clue showed that auxin had an essential role in regulating the formation of flower primordium (Luo et al., 2018; Shi and Vernoux, 2022). The explanation for auxin regulation on SAM differentiation is mainly due to the unbalance of auxin concentration in different zones, which was affected by different auxin transporter systems such as PIN protein and AUX/LAX influx carrier (Abley et al., 2016). Some models such as the flux-based and up-the-gradient models have been advised to explain the transportation and allocation of auxin in different cell zones (Bilsborough et al., 2011). However, the elaborate modulation system on SAM differentiation by auxin is still on the way.

In addition to genetic controlling SAM differentiation, environmental and agronomic practices such as low temperature and nitrogen application also have important roles in the differentiation (Olas et al., 2019). Although nitrogen application can affect crop growth and development, there are few evidence of the relationship between nitrogen application and SAM differentiation (Olas et al., 2019). It is well known that there is a close relationship between nitrogen application rate and auxin biosynthesis through the tryptophan pathway (Zhao, 2011; Fu et al., 2020; Gu et al., 2022). And auxin has important regulatory role in SAM differentiation. Therefore, our hypothesis in this research is that nitrogen application rate affects SAM differentiation through auxin mediation. In the present study, we first analyzed the effect of nitrogen application rate on the timing of SAM differentiation in rapeseed. And then, we dissected the auxin concentration in the SAM with various differential stages. Finally, we also assayed the gene expression of auxin biosynthesis, transportation, and SAM differentiation pathway through transcriptome. The research will enrich our scientific knowledge on the molecular regulation of SAM differentiation by nitrogen application rate through auxin regulation.

## Materials and methods

### Plant materials and experimental design

The field trials were conducted during the growing seasons of 2020–2021 and 2021–2022 at the Zhejiang Academy of Agricultural Sciences, Hangzhou, China. Commercial rapeseed (*Brassica napus* L.) variety zheyou 50 was used as plant material. The soil type in the experimental station is loamy clay (loamy, mixed, and thermic Aeric Endoaquepts). The previous crop was rice. Before sowing, fertilizers including calcium superphosphate, potassium oxide, and borax were manually applied at the rate of 375, 120, and 15 kg ha^−1^, respectively as a basal fertilizer dose. Approximately five to six rapeseed seeds were directly sown into the soil in each shallow hole at a depth of approximately 3 cm in each plot. After sowing, the plot was covered by a layer of soil and then sprayed 750 mL of s-metolachlor (96%, v/v) with 1,500 kg of water per hectare to control the weed. The excessive seedlings were removed and thinned to one plant in each hole after 1 month. The field was not irrigated during rapeseed growing seasons. The growth condition, mainly including mean temperature and rainfall, was illustrated in Supplementary Figure S1.

### Experimental design

Nitrogen fertilizer (urea) was applied before sowing with three levels, which were low nitrogen level (N1, 120 kg ha^−1^), optimal nitrogen level (N2, 240 kg ha^−1^), and high nitrogen level (N3, 360 kg ha^−1^). Under each treatment, 70% of the urea was applied into the soil before sowing and 30% of the urea was applied on top during the budding stage. A completely randomized block method was used with three replicates and the area of each block was 18 m^2^. The density of plants in each treatment was 120,000 plants ha^−1^ with the same management during the whole growing season.

### Identification and determination of floral meristem (FM) initiation time and morphology and sampling

**Figure 1:**
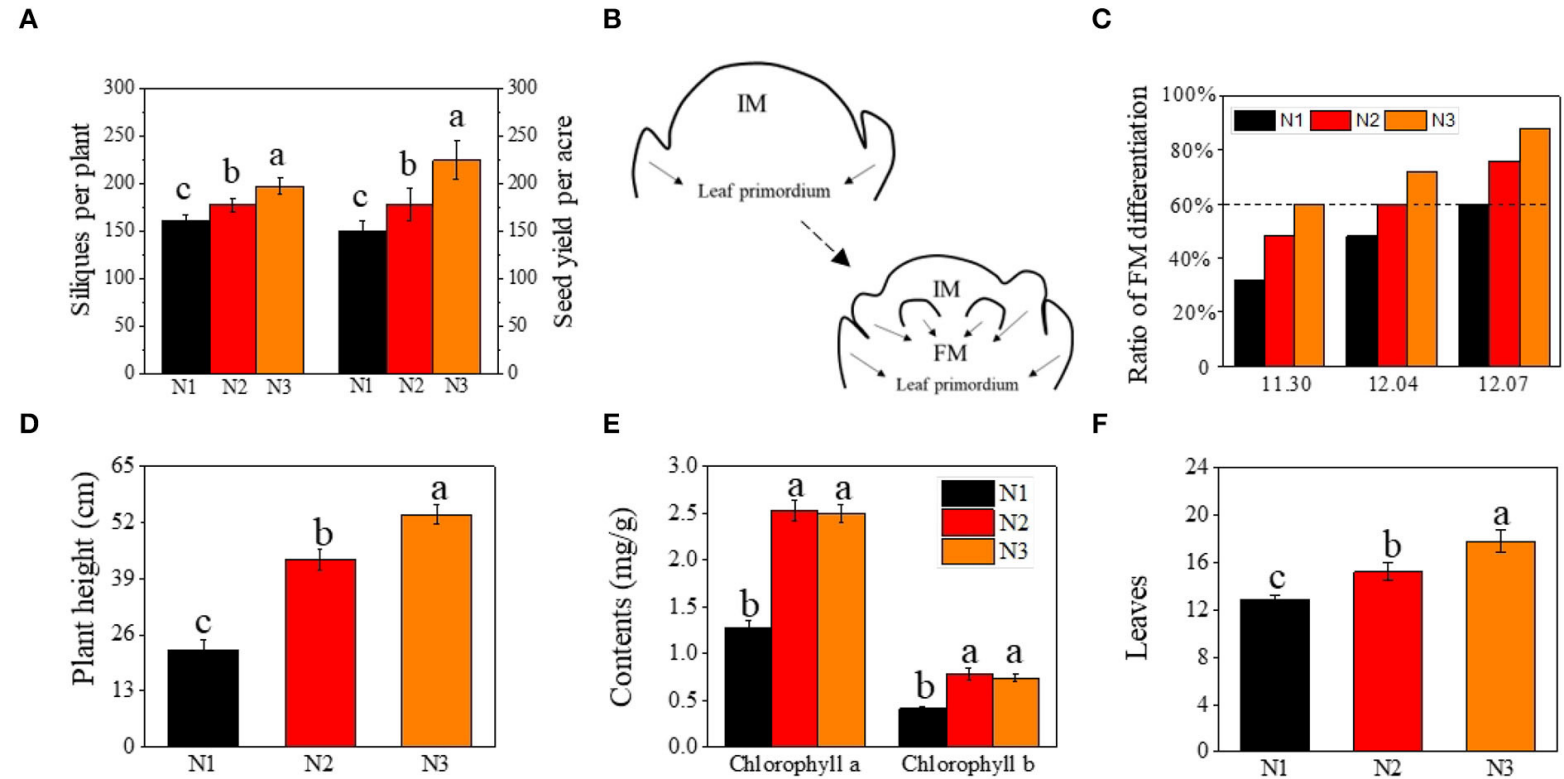
Effects of low N (N1), normal N (N2), and high N (N3) rates on silique number, and yield of rapeseed, floral meristems differentiation, plant height, chlorophyll contents, and leaves. **(A)** silique number and seed yield per acre; **(B)** the morphogenesis of floral meristems initiation; **(C)** ratio of floral meristems differentiation (%) under different N rates at different sampling stage; **(D–F)** represent plant height (cm), chlorophyll contents (mgg^−1^), and leaves, respectively; FM represented floral meristem. IM represented inflorescence meristem. Error bars represented SD value. Different lower cases represented significance at *p* ≤ 0.05.

About 30 days before the initiation of floral meristem differentiation, all rapeseed plants at a random area of 2 m^2^ in each plot were sampled (about 25 plants per plot) and the morphology of SAM was observed after peeling off all the visible blades under a stereoscopic microscope (VHX-950F, Japan) every 5 d (Zhang et al., 2016). The appearance of ball-shaped flower meristems (FM) in inflorescence meristems (IM) is determined as the initiation of FM differentiation of each plant, and 60% of plants entering initiation were determined as FM differentiation (FMd) time (Figures 1B,C).

The morphology of SAM at 5 days before N3 came into FMd (T0, November 25th), N3 just came into FMd (T1, November 30th), N2 entered into FMd (T2, December 4th), and N1 entered into FMd (T3, December 7th) and 5 d after N1 entered into FMd (T4, December 12th) were observed under scanning electron microscopy according to Vernoux et al. (2000), and the samples of SAM at these five stages were collected and kept at −80°C for RNA-seq and plant hormone determination.

### RNA extraction, library construction, sequencing, and analysis

Samples of SAM at T0, T1, T2, T3, and T4 stages under three nitrogen application levels were used as materials and total RNA was extracted using the polysaccharide and polyphenol total RNA isolation kit (Bioteke, Beijing, China). The libraries were constructed according to the manufacturer's instructions and performed on Illumina Nova-seq 6,000 system for sequencing, and HISAT2 was used to align all clean reads against the reference genome *Brassica napus* (Fu et al., 2018). The expression levels of genes were determined according to FPKM values. |log_2_FoldChange|>1 and *P*-value ≤ 0.05 were determined as differentially expressed genes (DEGs) using the DEseq2 methods and edgeR program (Liu et al., 2020). Genes related to auxin biosynthesis, auxin signaling transduction, and floral meristem differentiation were focused. The expression of each gene at 5 stages × 3 nitrogen levels were demonstrated as a heatmap after data centralization and standardization by R programming language.

### Determination of auxins contents by ultra-performance liquid chromatography-tandem mass spectrometry (UPLC-MS/MS)

Auxin contents of SAM at T0, T1, T2, T3, and T4 stages under three nitrogen application levels were determined through UPLC-MS/MS methods. The pretreatment of samples was performed according to Li et al. (2016). Supernatants were subjected to UPLC-MS/MS system, and then Multiple Reaction Monitoring (MRM), Analyst v.1.6.3, and MultiQuant v.3.0.3 were utilized to quantitate and quantify the raw data (Šimura et al., 2018). The contents of each auxin at 5 stages × 3 nitrogen levels were shown as a heatmap after data centralization and standardization by R programming language.

### Statistics

Data analysis was performed using IBM SPSS v.22.0 statistical software. Duncan's multiple range test (DMRT) was used to evaluate significant treatment effects at the significance level of *P* ≤ 0.05.

## Results

### Higher nitrogen application rate promoted the differentiation of FM and yield

The number of effective siliques and yield were significantly improved by the nitrogen application rate (Figure 1A). The number of effective siliques was 198 per plant on average under N3, which was 11.1 and 22.6% higher than under N2 and N1, respectively. The yield was also increased by 26.1 and 50.5% as compared to N2 and N1.

Correlation analysis showed that the ratio of FM differentiation was highly correlated with seed yield, with the correlation coefficient reaching 0.978, 0.992, and 0.977 at three sampling stages, respectively (Supplementary Figure S2). Given the close correlation between the timing of FM differentiation (FMd) and yield (effective siliques), we analyzed the timing of FM differentiation under different nitrogen application rates. Under optimal nitrogen rate (N2) treatment, FM differentiation of rapeseed initiated on December 4th (T2), 3 days earlier than under low nitrogen rate (N1), which initiated on December 7th (T3), and 4 days later than under high nitrogen rate (N3), which initiated on November 30th (T1) (Figure 1C).

In addition to the differences in the timing of the transition from vegetative to reproductive, plant height, leaf chlorophyll content, and leaf numbers were also analyzed when plants entered FMd under N3 treatment. As the nitrogen application rate increased, plant height under N3 was 24.2% and 139% higher than that under N2 and N1, respectively (Figure 1D). As for leaf chlorophyll a and chlorophyll b content, there were no significant differences between N2 and N3 treatments, however, both nitrogen application rates were significantly higher than that under N1 treatment, which increased by 98.8 and 90.2% under N2 treatment, and 96.3 and 79.8% at N3, respectively, as compared to N1 treatment (Figure 1E). For the number of leaves, it increased by 16.9 and 38.0% under N3 treatment when compared with N2 and N1, respectively (Figure 1F).

### Morphology of FMd under different nitrogen application rates

**Figure 2:**
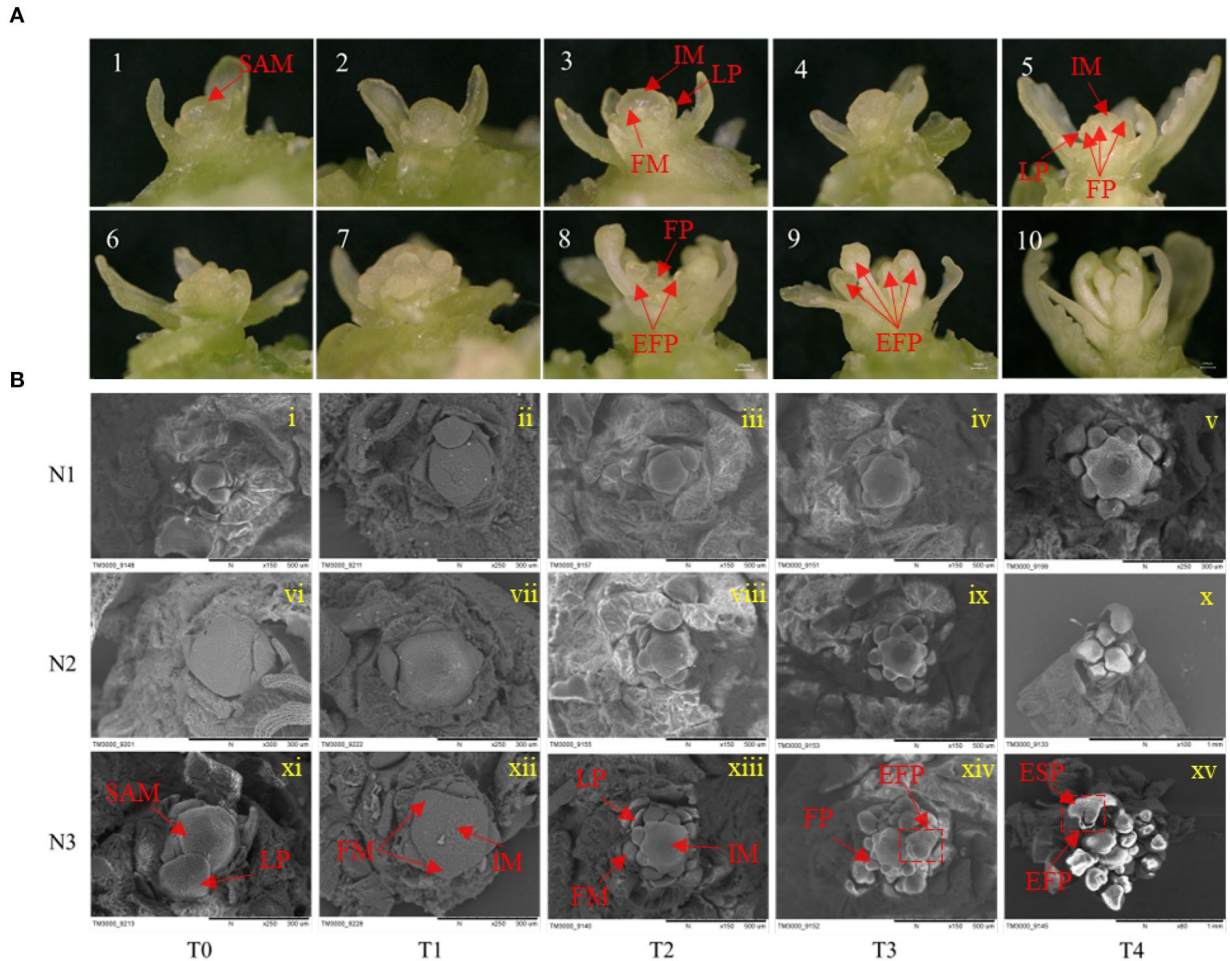
Effects of low N (N1), formal N (N2), and high N (N3) rates on the morphology of shoot apical meristems and floral meristems. **(A)** display of 10 consecutively different images of the development of rapeseed inflorescence and floral meristems under a stereoscope. **(B)** Scanning electron microscope images of inflorescence and floral meristems at T0, T1, T2, T3, and T4 sampling stages under low N (N1), normal N (N2), and high N (N3) rates. EFP, elongated floral primordium; ESP, elongated sepal primordium; FM, floral meristem; FP, floral primordium; IM, inflorescence meristem; LP, leaf primordium; SAM, shoot apical meristem.

**Figure 3:**
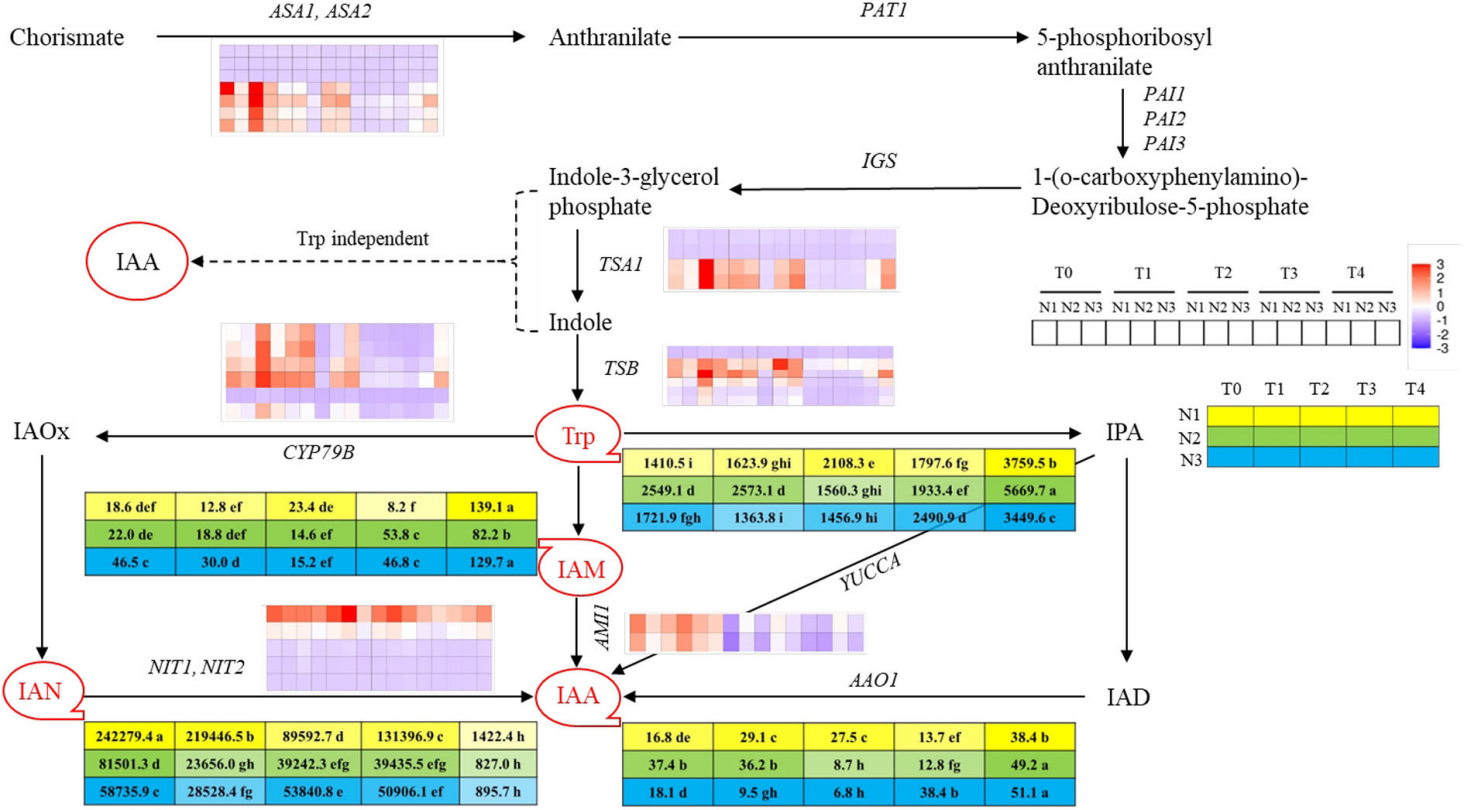
Overview of auxin and derivatives and genes involved in auxin biosynthesis pathway at T0, T1, T2, T3, and T4 stages under low N (N1), normal N (N2), and high N (N3) rates. IAA, Indole-3-acetic acid; IAN, 3-Indole acetonitrile; IAM, 3-Indole acetamide; Trp, tryptophan. Red circles with tails represented different auxin-derivatitves and the 5 × 3 matrix represented the contents under 3 N rates at five sampling stages. Color bars represent the trends of expression levels after centralization and standardization by the R programming language, red color represents higher expression, while purple represents lower expression relatively. Tables near Trp, IAM, IAN, and IAA represent each content (ngg^−1^) at T0, T1, T2, T3, and T4 stages under N1, N2, and N3 rates, and yellow, green and blue colors represent different N rates, while the color shades reflected the level of the contents. Dash lines indicated the supposed pathway of auxin biosynthesis while the solid line showed the detected pathway of auxin biosynthesis. Lower case behind the numbers in the tables represented significant differences, *p* ≤ 0.05.

For the morphological changes during FMd, the process of FMd was first imagined by stereomicroscope. One set of the process was illustrated in Figure 2A. The process can be roughly divided into five stages. In brief, in stage 1, plants were before floral meristem differentiation with leaf primordia (LP) around the SAM (Figure 2A, 1~2). Stage 2, SAM developed into inflorescence meristems (IM) quickly, and plants were entering floral meristem differentiation with one or two protrusion(s) on the IM (Figure 2A, 3). Stage 3, floral primordium burst around the IM (Figure 2A, 4~7). Stage 4, floral primordial rapid accumulation corresponded with the floral organ development (Figure 2A, 8). Stage 5, the main inflorescence was established (Figure 2A, 9~10).

To further understand the morphological differences in the timing of the transition from vegetative to reproductive growth caused by different nitrogen application rates, a scanning electron microscope was used to have a clear presentation. At the T0 stage, the SAM was not differentiated with only leaf primordia under three nitrogen application rates (Figure 2B i, vi, and xi). At the T1 stage, SAM developed into IM with several bulbs surrounded under N3 treatment, suggesting the initiation of FM differentiation. However, there were no obvious protrusions under N1 and N2 treatments (Figure 2B ii, vii, and xii). At the T2 stage, there were about eight bulbs observed around the IM under N3 and two protrusions under N2 treatment, while, no protrusion was found around the IM under N1 treatment at this stage (Figure 2B iii, viii, and xiii). At the T3 stage, many bulbs were distributed around the SAM and some bulbs started to develop into flowers under N3 treatment, while considerable bulbs were around the IM but no differentiated bulbs under N2 treatment. There were two bulbs observed around the IM under N1 treatment indicating the start of FM differentiation at this stage (Figure 2B iv, ix, and xiv). At the T4 stage, rapeseed entered FM differentiation under all nitrogen application rates only with the different differentiation rates (Figure 2B v, x, and xv). The morphologic result clearly showed that a higher nitrogen application rate accelerated the timing of the transition from vegetative to reproductive growth in rapeseed.

### Endogenous auxin contents in rapeseed SAM were influenced by different nitrogen application rates

To uncover the reason for earlier timing of the transition from vegetative to reproductive growth promoted by higher nitrogen application rate, the variation tendency of endogenous auxin contents of SAM at five stages under three nitrogen rates were assayed. By utilizing UPLC-MS/MS method, four auxin metabolites involved in auxin biosynthesis were detected, which were tryptophan (Trp), 3-Indole acetonitrile (IAN), 3-Indole acetamide (IAM), and Indole-3-acetic acid (IAA) involving in two auxins biosynthetic pathways, respectively, which were indole acetaldehyde oxime pathway (IAOx pathway) and indole acetamide pathway (IAM pathway). Among those products, Trp and IAN contents were dominant as compared to other ones. The contents of Trp were higher before each FM initiation stage under different nitrogen levels, then decreased sharply at the FMd stage and gradually increased whereafter. The Trp content at N3-T1 was significantly lower than that at N1-T3 (both at the FM initiation stage), which showed a 24.1% decrement of content. However, no significant difference was found between N2 and N3 or N1 and N2, at each FM initiation stage. The contents of IAN were dramatically higher than other IAA derivatives and showed a trend of decline with fluctuation, while the contents of IAN at T4 were largely decreased than other stages. Aimed at the FM initiation stage, IAN content at N1-T3 was 234.8 and 360.6% significantly higher than those at N2-T2 and N3-T1. The contents of IAM involved in the IAM pathway first showed a decreasing trend in each FMd stage under each nitrogen application rate, and then went up. In terms of the IAM contents at each FM initiation stage, the value under N3 was significantly higher than N2 and N1, with increments of 105.5 and 265.9%. IAA was biosynthesized under the combined action of IAN and IAM, the contents of which were minimized at FMd under each nitrogen application rate. After FMd, the content of IAA then rose and reached the highest value at the T4 stage under three nitrogen application rates. Aimed at the FM initiation stage, IAA content at N3-T1 was 30.7% significantly lower than that at N1-T3, while showing no difference compared to N2-T2 (Figure 3).

### Temporal changes of gene expressions involved in auxin biosynthesis, auxin signaling transduction, and FM differentiation

To further reveal the molecular mechanism of the regulation of advanced timing of the transition from vegetative to reproductive stage under a higher nitrogen application rate, the RNA-seq analysis was performed for samples with different FMd stages as mentioned previously. The analysis of the annotation, differentially expressed genes, and gene ontology was listed in Supplementary Tables S1–S3. However, the current study only focused on the auxin metabolism, therefore, the expression of the genes only correlated with auxin biosynthesis, signaling transduction, and FM differentiation was assayed, and all of them were listed in Supplementary Tables S4–S6.

In total, seven kinds of auxin biosynthesis genes were identified, which were *ASA1/ASA2, IGS, TSA1, TSB1, CYP79B, NIT1/NIT2*, and *AMI1*, and the general expression modes were shown in Figure 3. In the IAOx pathway, six copies of *CYP79B* gene were identified in this study, which is the key gene functioning on the conversion of Trp to IAOx. There was one copy (*BnaC03G0681300*) that showed downregulation mode throughout the FMd stages under all nitrogen application rates. However, the other five copies of *CYP79B* showed higher expression levels at the first three stages, with the expression level at N3 obviously higher than at N2 and N1, respectively, then declined rapidly. *NIT* was the second key gene in the IAOx pathway which functions on the conversion of IAN to IAA. There were five copies of *NIT* identified in the study. Among those genes, *BnaC02G0078200* showed a strong upregulation mode, and the expression level of which reached the highest at each FM initiation stage under different N application rates. However, *BnaA02G0068000* showed a very weak upregulation mode and the remaining three copies showed downregulation mode under three nitrogen application rates. *AMI1* involves in the IAM pathway and functions in transferring IAM to IAA. There were two copies of *AMI1* found in the study. Both copies showed higher expression levels with upregulation mode at the first two stages (T0 and T1) and then also decreased to a relatively low level with downregulation mode (Figure 3). The expression of genes encoding key enzymes before the pathway of auxin biosynthesis including *ASA1/ASA2, IGS, TSA1*, and *TSB1* were also analyzed. Among those genes, *IGS* was not identified in this study. There were seven copies of *ASA1/ASA2* identified in this study. However, *BnaA03G0229800, BnaC03G0270600*, and *BnaC03G0270700* showed downregulation mode during the FMd stage under all nitrogen application rates. The remaining four copies showed strong expression under at T0 to T2 stages of FMd, while generally showing downregulation mode from the T3 stage. Furthermore, at the T0 stage, the highest expression level was found in N3 treatment. As for *TSA1*, there were four copies identified. Two of them (*BnaA09G0026900* and *BnaC09G0011700*) showed downregulation mode throughout the FMd stage under all nitrogen application rates. The other two copies, *BnaA09G0505300* and *BnaC08G0346300*, generally showed upregulation mode from T0 to T2 under three nitrogen application levels. Furthermore, both genes showed the highest expression levels at N3 in T0, T2, and T4 FMd stages (Figure 3).

**Figure 4:**
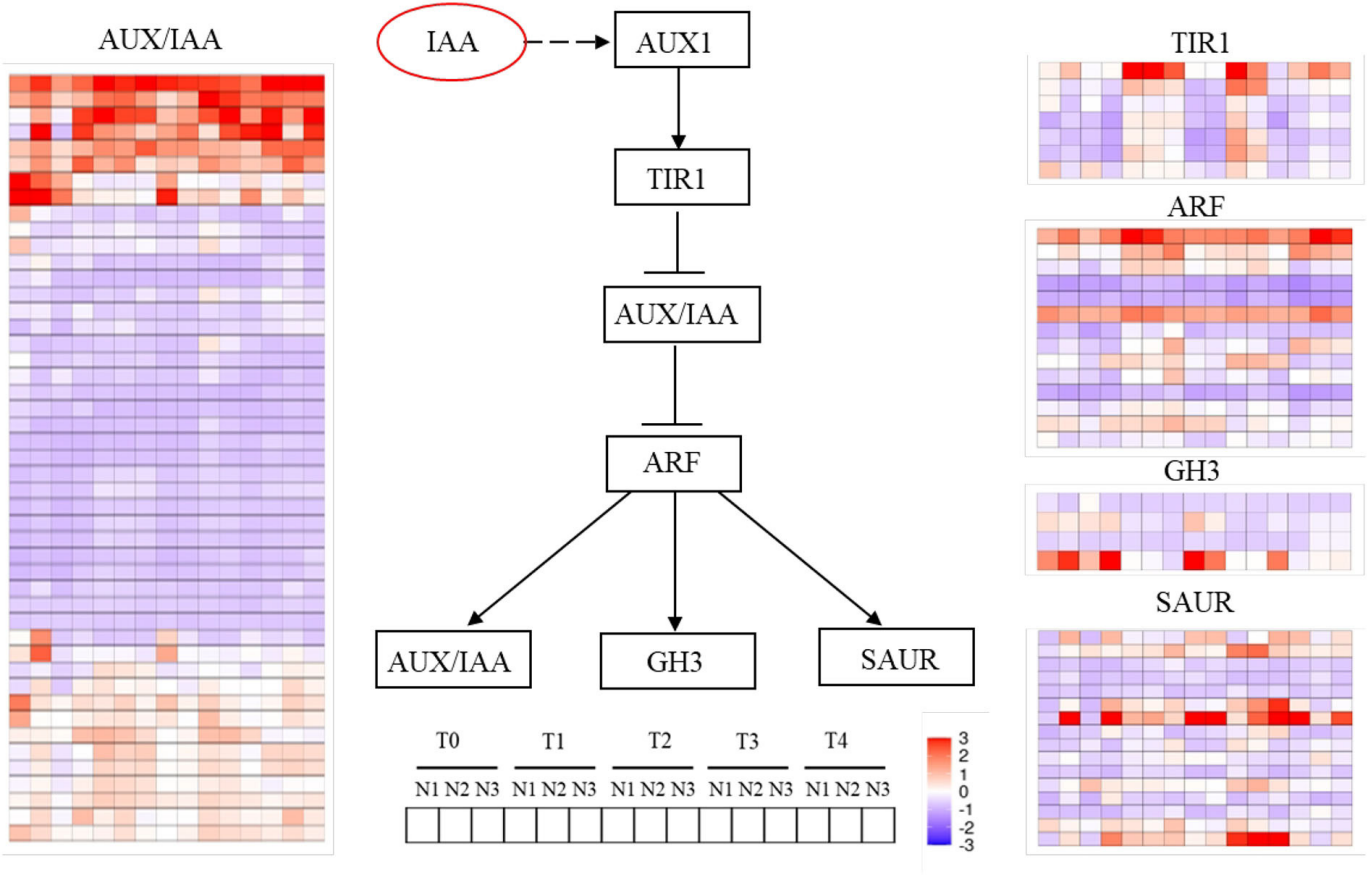
Changes of genes involved in auxin signaling transduction at T0, T1, T2, T3, and T4 sampling stages under low N (N1), normal N (N2), and high N (N3) rates. *ARF, Auxin response factor*; *AUX1, Auxin influx carrier 1*; *AUX/IAA, Auxin responsive protein*; *TIR1, Transporter inhibitor response 1*. Color bars represented the trends of expression levels after centralization and standardization by the R programming language and red color represents higher expression, while purple represents lower expression relatively.

There were totally five kinds of auxin signaling transduction genes identified, which were *TIR1, AUX/IAA, ARF, GH3*, and *SAUR*. *AUX/IAA* genes are a large family in plants and there were 47 genes found in the study. The genes can be divided into three types according to the expression levels. The first group was a strong expression with upregulation mode, which is composed of eight genes generally throughout the FMd stage under three nitrogen application rates. The second group was a weak expression with upregulation, which had 12 genes. And the third group was downregulation, which had 27 genes. Among the eight strongly expressed *AUX/IAA* genes, the expression levels at T1 and T3 were much stronger than in other stages. As for the nitrogen application rate, N2 had higher expression levels than N1 and N3 in general. A similar expression trend of the weak upregulation genes was observed. As for *TIR1*, there were seven genes found in the study. These genes had similar expression mode, which was the higher expression levels at T1 and T3 FMd stages. Furthermore, higher expression levels at N2 and N3 were found in all genes at the T1 FMd stage. However, at T2 and T3 FMd stages, the reverse trend, that is, higher expression levels of all genes at N1 was observed. At the T4 FMd stage, only *BnaA03G0267400* showed upregulation in all genes under three nitrogen application rates. As for *ARF*, there were two genes, *BnaA01G0267900* and *BnaC01G0328100*, that showed strong expression with upregulation, especially at T1 and T4 stages. Five *ARF* genes with downregulation mode were identified, however, *BnaA07G0137300, BnaA08G0250200*, and *BnaC05G0168800* had very strong downregulation mode in all FMd stages and nitrogen application rates. Generally, the N2 treatment had higher expression levels than in other nitrogen application rates. As for *GH3*, there were four genes identified in the study. Two genes, *BnaA03G0053200* and *BnaA09G0587900*, were in the downregulation mode nearly in all FMd stages and nitrogen application levels. One gene, *BnaC09G0017400*, had a strong expression with upregulation mode at most FMd stages. However, there was no obvious expression trend for responding to three nitrogen application rates at each differentiation stage. For *SAUR*, there were 16 genes identified in the study. Among them, seven genes showed upregulation mode at most FMd stages under three nitrogen application rates, and one gene, *BnaC01G0155600*, with relatively strong expression pattern. The gene had very strong expression levels at T2 and T3 stages under N3 treatment (Figure 4).

**Figure 5:**
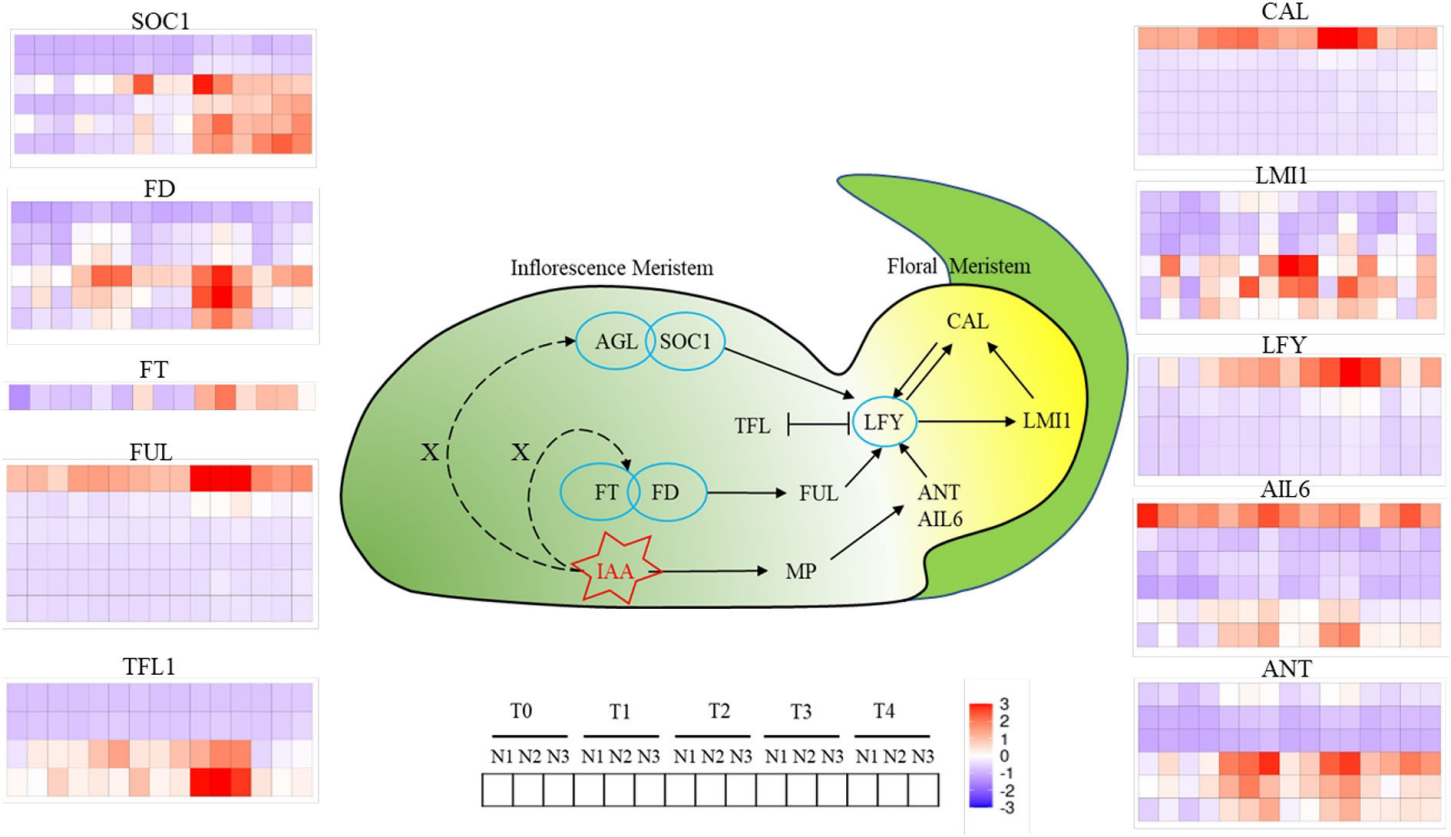
Changes of genes involved in floral meristems identity genes at T0, T1, T2, T3, and T4 stages under low N (N1), formal N (N2), and high N (N3) rates. Color bars represented the trends of expression levels after centralization and standardization by R programming language and red color represents higher expression, while purple represents lower expression relatively.

A total of 10 kinds of genes related to FM differentiation were identified, which were divided into two categories, which were signaling integration (*SOC1, FD, FT, FUL, TFL1*) and FM identity (*CAL, LMI1, LFY, AIL6, ANT*). As for *SOC1*, six copies were identified and two copies were in the downregulation mode throughout all FMd stages under three nitrogen application rates. The other four copies had an increasing trend of expression levels from T0 and higher expression levels at the T3 and T4 stages. As for *FD*, six copies were identified as well. Three copies were mainly in the downregulation mode during FMd stages and three nitrogen application rates. However, the other three copies were strongly expressed at the T3 stage with the highest expression amount at N2 treatment. One *FD* copy, *BnaA03G0552500*, showed a relatively higher expression amount with upregulation mode at T1, T2, and T4 in addition to T3. Furthermore, higher expression levels were found under N2 and N3 than that under N1 at T1 and T4. As for *FT*, there was only one copy detected in the study. The expression level was in the increasing trend during the FMd stage. At the T2 stage, the expression level of *FT* was in the upregulation mode at N1 while that was in the opposite mode at N2 and N3. However, the strongest expression of the gene was at N2 at T3. Although there were six copies identified for *FUL*, only one copy, *BnaA03G0404700*, showed upregulation mode. The highest expression level was found at the T3 stage and small differences in the expression level were found among three nitrogen application rates. For *TFL1*, there were four copies identified in the study, and two copies were in the downregulation mode throughout the FMd stage and all nitrogen application rates. Two copies, *BnaA03G0012400* and *BnaC03G0016500*, showed very strong expression at the T3 stage. Furthermore, *BnaA03G0012400* had the highest expression amount at T1 to T3 at N3. In the FM identity gene systems, there were six and four copies identified in the study for *CAL* and *LFY*, respectively. However, only one copy of both genes, *BnaC02G033300 (CAL)* and *BnaC03G0561300 (LFY)*, respectively, showed strong expression with up-regulation mode at all FMd stages and nitrogen application rates. For *BnaC02G0233300* (*CAL*), the expression level at T3 was the highest followed by T1. However, the expression levels at N1 and N2 were much higher than that of N3 at the T3 stage, while the reverse trend was found in T1. For *LFY*, the expression level was increased from T0 to T3. The highest expression level of *LFY* was at T3 and N2 treatment. There were six copies identified for *LMI1, AIL6*, and *ANT*, and all of them showed three copies mainly having upregulation mode during FMd stages and three nitrogen application rates. For *BnaA10G0288900* (*LMI1*), the highest expression level was found at T2 with N2 treatment which was in accordance with the timing of FMd at this stage. As for *BnaC09G0552500* (*AIL6*), there were no relatively small differences in expression levels among FMd stages and nitrogen application rates. As for *BnaA01G0012600, BnaA03G0565600*, and *BnaC07G0540500* (*ANT*), all of them showed the highest expression levels at T1 and N3, T2 and N1, and T3 and N2 treatments, respectively (Figure 5).

## Discussion

### Accelerating the initiation of FM differentiation by a higher nitrogen application rate is an effective way for yield increasing

FM differentiation is the beginning of the transition from vegetative to reproductive growth of flowering plants. The initiation time and differentiation ability of FM determine the number of flower buds formed, which is of great significance to the establishment of rapeseed yield (Wrucke et al., 2019). The initiation of FM differentiation occurs at the SAM, and the earlier FM initiation time will enhance the efficiency of rapeseed utilizing the late autumn temperature and light resources, which can promote reproductive growth a lot (Fiebelkorn and Rahman, 2016).

In this research, we found that the initiation time of FM was largely regulated by different based-N-fertilizer levels. The FM initiation time was at December 7th under low N level while with increase of N fertilization, the FMd could be promoted by 3 to 7 days. The advanced dates can strive temperature and light resources of late-autum a lot for rapeseed growth and hence the increase of siliques and yield up to 22.6 and 50.5%, respectively (Figure 1). To determine if the increase in yield was related to the initiation and development of FM, a scanning electron microscope was utilized for the observation and identification of FM morphology. The results showed that the appearance of floral meristems, sepal primordium, gynoecium, and androecium primordium occurred earlier in higher N rates than in lower. What's more, the emergence of FMs enlarging to ball-shaped structures and the separation of FMs from IM also went earlier (Figure 2), which accelerated the establishment of reproductive organs, hence, more temperature and lights were utilized.

### Trp-IAOx-IAN-IAA biosynthesis pathway might be the major auxin biosynthetic pathway in rapeseed SAM, and the decrease of endogenous auxin contents may be the essential condition for FM initiation

FM differentiation, as the transition from SAM to floral meristem, is induced by a series of environmental signal pathways, including photoperiod, low temperature, and carbohydrates, and is accompanied by a large number of physiological, biochemical, and gene expression changes. Studies have shown that plants have already started the regulation and transformation of the physiological and molecular mechanisms before the morphological change of SAM (Liu et al., 2009). Therefore, illuminating the changes in physiology, biochemistry, and gene expression in different processes before and after FM differentiation is of great significance for explaining the mechanism of FM differentiation, shortening the growth period, and improving the yield of rapeseed (Yan et al., 2021).

The homeostasis of endogenous hormones, as the basis of plant vegetative growth and reproductive growth, plays an important role in the flowering process of plants and is also one of the important conditions for flowering induction (Daviere and Achard, 2016; Castorina and Consonni, 2020). Auxin as a major phytohormone controls numerous aspects of rapeseed development and coordinates the responses to the environment, and so does the initiation of FM and organs development (Brumos et al., 2018). However, the role of how auxin determines the development and transition of IM to FM remains less mechanistically. According to previous studies in *Arabidopsis*, auxin was accumulated at the position of FM and gradually decreased with the extension of distance, and it is the gradient that contributes to the development and differentiation of floral organs (Benková et al., 2003). However, the changes in auxin contents in SAM and the gene expression in its biosynthetic pathway in different periods are not clear. In this study, we identified the contents of auxins at five stages under three different N levels of SAM through UPLC-MS/MS, and four auxins were detected, which involved two auxin biosynthetic ways, which were IAOx way and IAM way, respectively. Previous research showed that the indole pyruvic acid (IPA) way is the main auxin biosynthetic way, while IAOx and IAM contribute less (Mashiguchi et al., 2011; Won et al., 2011). However, we detected a large amount of auxin biosynthetic precursors in IAOx and IAM ways, which were IAN and IAM, and the contents of IAN were 1,740 times of IAM in terms of the maximum for each other, while no auxins involved in IPA way were detected, which indicated that Trp-IAOx-IAN-IAA might be the major auxin biosynthetic pathway in rapeseed shoot apical meristems. What's more, we found that both of the contents of Trp and IAA showed a trend of increase in the first and then a sharp decrease in the FM initiation stage followed by a gradual rise (Figure 3). Through the combination of the images of SEM, we presumed that IM needs relatively low auxin contents as the signal to initiate the differentiation of FM, then accumulated more auxin contents for the following up development of FMs, and so did the sepal primordium. At the same time, we found that the expression of genes such as *TSA1, TSB1, CYP79B, NIT*, and *AMI1* was higher expressed in 1 to 2 stages earlier than each FM initiation stage, while the maximum value came earlier under high N level than under low N level, which indicated that high N rate accelerates the activity of auxin biosynthesis genes, while this acceleration occurred 1 to 2 stages before FM initiation.

### Auxin signaling transduction and FM identity genes were largely regulated under different nitrogen levels

Interactions of endogenous growth and developmental signals with gene expressions constitute a complex pathway for plant reproductive growth induction. We identified that most of the auxin signaling transduction genes were upregulated in T1 to T3 stages, relative to one stage later than auxin biosynthesis, while the higher expression of FM differentiation-related genes came at the last two stages.

Among them, *AGL24, SOC1, FT, FD*, and *FUL* functioned as integrating environmental and other signals in SAM and transferring the signal to *LFY* for *FM* identify and specification (Liu et al., 2009). SOC1 is highly expressed in IMs and induces the expression of LFY by binding to the promoter region of *LFY* through the AGL24-SOC1 protein complex (Li et al., 2008). FT-FD complex also promotes the activity of SOC1 or FUL in IMs, hence the expression of IM identity genes such as LFY and CAL were enhanced (Li et al., 2008; Liu et al., 2008). According to our research, the expression of *SOC1* and *FD* began to rise at the T1 stage under the N3 level, while the significant upregulation occurred 1 to 2 stages later than N3 under N1 and N2 levels (Figure 5). Terminal flower 1 (*TFL1*) as a repressor of flowering, is higher expressed in SAM, while less expressed in FM, which functions by maintaining the disordered growth of IM and inhibiting the expression of *LFY* in IM (Schiessl et al., 2017; Zhang et al., 2018). In this study, we can find that, under normal or high N rate, the expression of *TFL1* was repressed until the T4 stage, while the upregulation occurred two stages earlier under a low N rate compared with N3, which indicated that the inhibition of *TFL1* on FM development was weakened under high N rate. *LFY*, as a central gene of FM, is reported to regulate the formation of FM through auxin signaling transduction. *ANT* and *AIL6* as the upstream genes of *LFY* functioned parallel and redundantly to regulate the expression of *LFY* and the response to auxins (Yamaguchi et al., 2016). According to our results, *ANT* and *AIL6* were upregulated at the T1 stage, 1 to 2 stages earlier than LFY, which was consistent with the previous research (Yamaguchi et al., 2016). At the same time, *CAL*, another distinctive FM-identified gene, was regulated by *LFY* directly or through *LMI1*, and the expression was relatively higher and earlier under a high N rate.

In brief, FM-identified genes were higher and earlier expressed under high N rate compared with normal or low N rate, while the activity of FM repressor was limited, which co-contribute to the advanced development of FM under high N rate.

## Data availability statement

The datasets presented in this study can be found in online repositories. The names of the repository/repositories and accession number(s) can be found below: https://www.ncbi.nlm.nih.gov/ - PRJNA846178.

## Author contributions

SH designed the experiment and revised the manuscript. PH investigated (all) and wrote the manuscript. BL analyzed the RNA-seq data. HH analyzed the auxin content measurement. BX and LH collected the agronomic data and microscopy imaging. All authors approved the final version of the article.

## Funding

This work was supported by the National Key Research and Development Project (2018YFD1000900), the Zhejiang Key Laboratory of Digital Dry Land Crops (2022E10012), and the Zhejiang Science and Technology Major Program on Agricultural New Variety Breeding (2021C02064).

## Conflict of interest

The authors declare that the research was conducted in the absence of any commercial or financial relationships that could be construed as a potential conflict of interest.

## Publisher's note

All claims expressed in this article are solely those of the authors and do not necessarily represent those of their affiliated organizations, or those of the publisher, the editors and the reviewers. Any product that may be evaluated in this article, or claim that may be made by its manufacturer, is not guaranteed or endorsed by the publisher.

## Supplementary material

Supplementary Figure S1 The growth condition of mean temperature and rainfall during the field trials.

Supplementary Figure S2 Correlation analysis between ratio of floral meristems differentiation, siliques per plant, and seed yield per acre. The values above the double-sided arrows represent the correlation coefficients on 30th Nov, 04th Dec, and 07th Dec, respectively.

Supplementary Table S1 Total differentiation expressed genes and GO, KEGG analyzed genes in the study with different nitrogen application rates and floral meristem differentiation timings.

Supplementary Table S2 Total upregulated and downregulated genes in the study with different nitrogen application rates and floral meristem differentiation timings.

Supplementary Table S3 Annotation of total differentiation expressed genes.

Supplementary Table S4 Genes were identified in correlation to auxin biosynthesis.

Supplementary Table S5 Genes were identified in correlation to auxin signaling transduction.

Supplementary Table S6 Genes were identified in correlation to floral meristem differentiation.
