# Supplementary material for: Auxin-regulated timing of transition from vegetative to reproductive growth in rapeseed (Brassica napus L.) under different nitrogen application rates
Source: Front Plant Sci. 2022 Sep 9;13:927662. doi: 10.3389/fpls.2022.927662 (PMC9501695; doi:10.3389/fpls.2022.927662)
Supplement: Supplementary Figure S1 — The growth condition of mean temperature and rainfall during the field trials. [file Presentation_1.PPTX]

## Slide 1
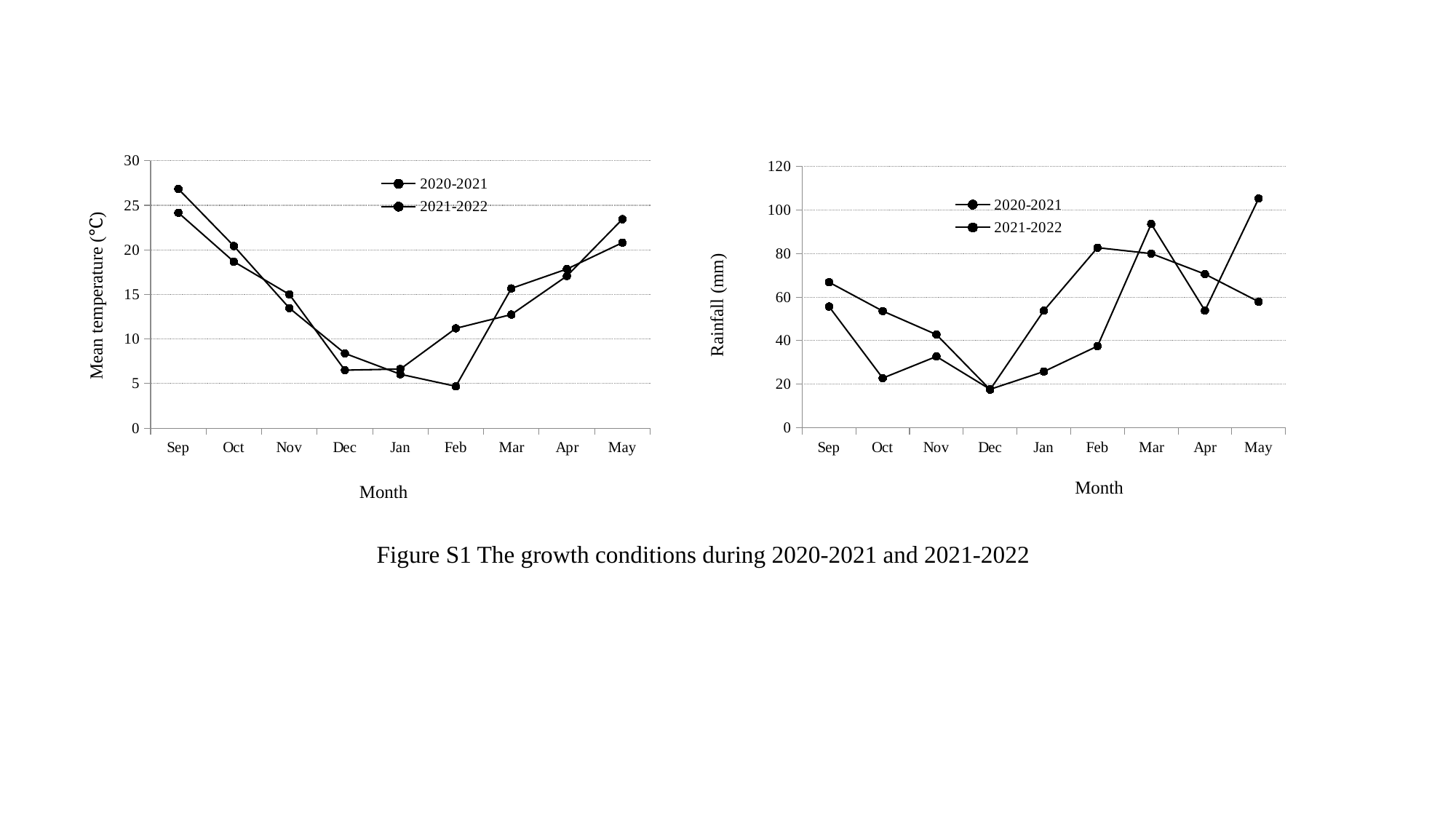

### Chart
| Category | 2020-2021 | 2021-2022 |
|---|---|---|
| Sep | 24.166666666666664 | 26.833333333333325 |
| Oct | 18.677419354838708 | 20.435483870967733 |
| Nov | 15.0 | 13.466666666666669 |
| Dec | 6.503528225806451 | 8.38709677419355 |
| Jan | 6.629032258064515 | 6.048387096774194 |
| Feb | 11.196428571428571 | 4.69642857142857 |
| Mar | 12.741935483870966 | 15.677419354838712 |
| Apr | 17.066666666666666 | 17.849999999999994 |
| May | 23.435483870967733 | 20.806451612903224 |
### Chart
| Category | 2020-2021 | 2021-2022 |
|---|---|---|
| Sep | 66.86 | 55.690000000000005 |
| Oct | 53.61 | 22.810000000000002 |
| Nov | 42.8 | 32.75 |
| Dec | 17.56 | 17.69 |
| Jan | 53.77 | 25.810000000000002 |
| Feb | 82.69 | 37.5 |
| Mar | 80.0 | 93.58 |
| Apr | 70.56 | 53.809999999999995 |
| May | 57.91 | 105.28 |Mean temperature (℃)
Rainfall (mm)
Month
Month
Figure S1 The growth conditions during 2020-2021 and 2021-2022

## Slide 2
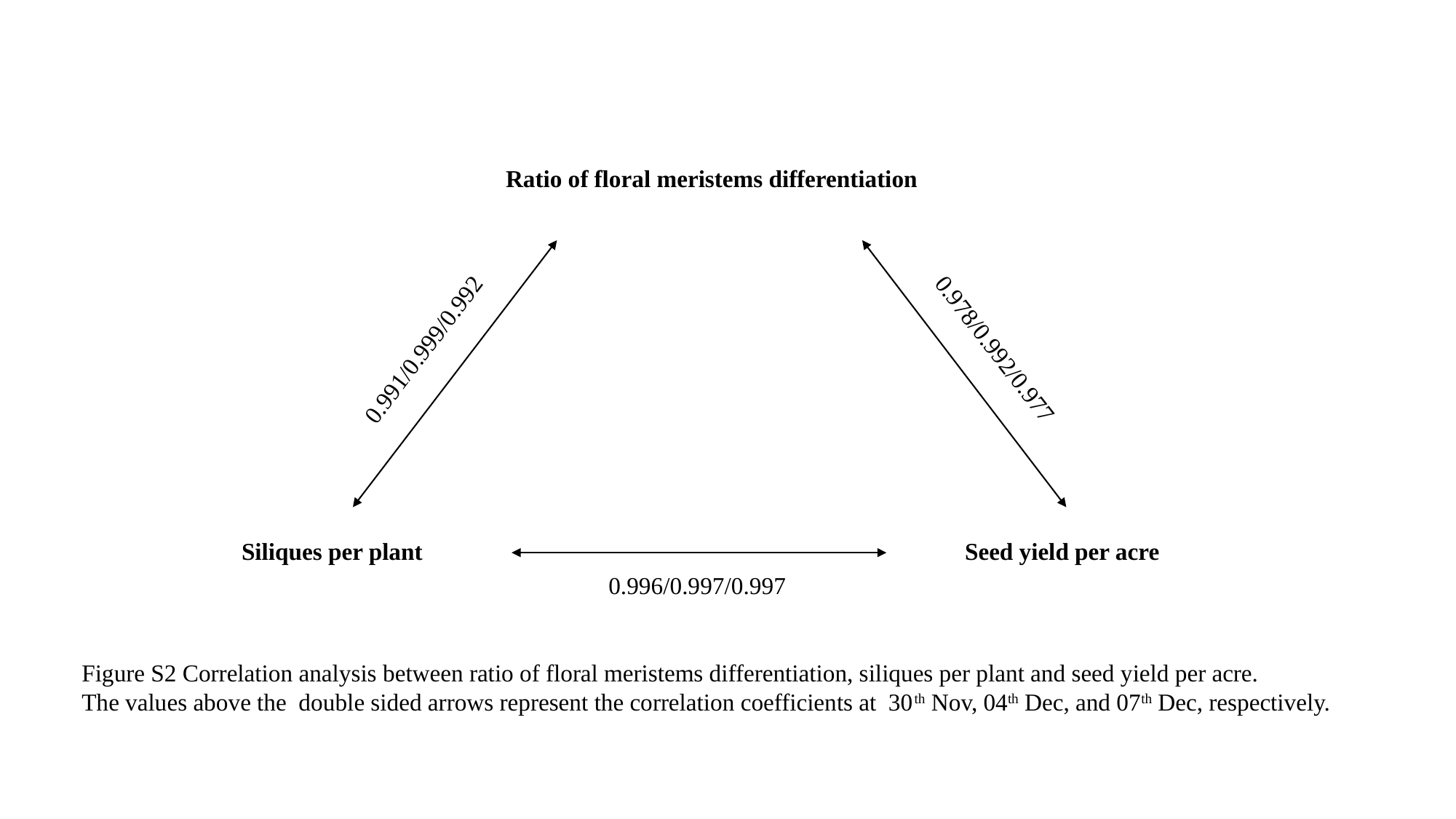

Ratio of floral meristems differentiation
0.978/0.992/0.977
0.991/0.999/0.992
Siliques per plant
Seed yield per acre
0.996/0.997/0.997
Figure S2 Correlation analysis between ratio of floral meristems differentiation, siliques per plant and seed yield per acre.
The values above the double sided arrows represent the correlation coefficients at 30th Nov, 04th Dec, and 07th Dec, respectively.
